# 0N4R Tau aggregates producing morphologically different and structurally similar “on-path” and “off-path” oligomers

**DOI:** 10.1039/d6cc02327d

**Published:** 2026-07-09

**Authors:** Joshua T. Skrehot, Mikhail Matveyenka, Dmitry Kurouski

**Affiliations:** a Department of Biochemistry and Biophysics, Texas A&M University College Station Texas 77843 USA dkurouski@tamu.edu; b Department of Biomedical Engineering, Texas A&M University College Station Texas 77843 USA

## Abstract

We utilized nano-infrared spectroscopy to resolve the morphology and structure of Tau aggregates. We identified “on-path” donut-like aggregates that evolve into fibrils and “off-path” round oligomers that do not. Despite morphological differences, both species share similar β-sheet-rich structures, providing critical insights into the mechanisms of Tau aggregation.

## Abstract 2

We utilized nano-infrared spectroscopy to resolve the morphology and structure of Tau aggregates.

## Graphical abstract

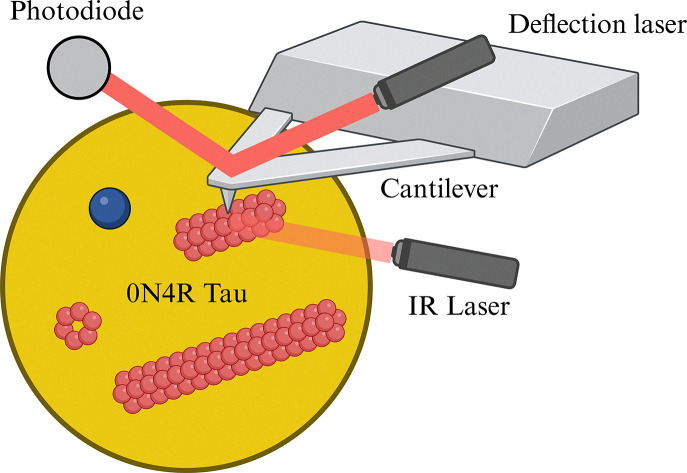

Microtubules form intracellular skeletons responsible for a large spectrum of critically important physiological functions, including metabolism, signaling, division, and motion. Assembly and disassembly of cell microtubules are determined by various isoforms of Tau that are produced as a result of alternative splicing of exons 2, 3, and 10.^1–4^ These isoforms can have zero (0N4R), one (1N4R), or two (2N4R) N-terminal inserts that enhance the binding of Tau isoforms to the tubulin of microtubules.^5–8^

Under pathological conditions known as Alzheimer's disease (AD) and tauopathies, Tau aggregates form spherical oligomers that later propagate into β-sheet-rich filaments known as amyloid fibrils.^9–12^ Although the secondary structure of Tau fibrils has been well understood, there is very little known about the structural organization of Tau oligomers.^12–15^ This is primarily due to their transient nature and high morphological heterogeneity.^13,16–19^ At the same time, pathological progression of AD has been attributed to the cell-to-cell transfer of Tau aggregates between anatomically connected neurons.^20–22^ Such cell-to-cell transfer has been demonstrated in numerous *in vitro* neuronal models.^20,23–25^

In the current study, we utilize innovative nano-infrared spectroscopy, also known as atomic force microscopy–infrared (AFM-IR) spectroscopy,^26–30^ to investigate the morphology and secondary structure of Tau oligomers formed at the early stages of protein aggregation. In AFM-IR, a metallized scanning probe can be positioned at the surface of individual protein aggregates.^31–34^ Next, infrared light is used to trigger thermal expansions in the sample that are reordered by the probe. These thermal expansions are converted into IR spectra, which, in turn, can be used to examine the secondary structure of protein aggregates.^35–37^ Using AFM-IR, we recently demonstrated that protein aggregation could yield structurally and morphologically diverse protein aggregates (α-synuclein and amyloid β)^34,38^ or two distinctly different types of oligomers (human islet amyloid precursor protein, hIAPP).^39^ In the former case, structural evolution from an antiparallel to a parallel β-sheet has been observed. In the latter case, the protein simultaneously formed donut-like (DO) and round (RO) oligomers that had drastically different secondary structures.^39^ It has also been shown that RO persisted throughout the entire course of protein aggregation, while DO quickly disappeared with the appearance of fibrillar species. Based on these findings, Warren and co-workers made a conclusion that DO were “on-path” oligomers that yielded fibrils, while RO were “off-path” species that generated no higher-order supramolecular ensembles.^39^

In this work, we investigated the morphology and secondary structure of protein aggregates formed by 0N4R Tau. This isoform lacks N repeats that can change the mechanisms of Tau aggregation.^15,40^ At the same time, it is important to note that the 0N4R Tau isoform has pathogenic significance in tauopathies.^16,18,41,42^ For this, sample aliquots were taken during the onset (8 h), during the middle (24 h), and at the end (48 h and 72 h) of protein aggregation (Fig. S1). Next, atomic force microscopy (AFM) was used to examine the topology of protein aggregates observed at all four time points (Fig. 1A). During the onset of protein aggregation (8 h), we observed the presence of both DO and RO. DO tended to exhibit lower heights than RO, although the difference was not statistically significant. We also found that during the early stages of Tau aggregation (24 h), DO disappeared, while only RO were observed together with fibrillar (FB) species (Fig. 1A). The same species (RO and FB) were also observed by AFM at the late stage of Tau aggregation (48 h and 72 h). It should be noted that RO observed at the early (8 h) and late (48 h) stages of protein aggregation were very similar in height (15–20 nm). These aggregates were also similar to fibrillar species observed at the middle (24 h) and late (48 and 72 h) stages of Tau aggregation (∼10 nm). These results suggest that DO observed during the onset of Tau aggregation were highly likely to produce fibrils. Thus, these aggregates are likely to be the “on-path” species of Tau aggregation. At the same time, our results suggest that RO are the “off-path” species that yield no higher-order protein aggregates. It should be noted that additional studies are needed to fully understand how DO propagate into fibrillar species. Finally, we emphasize that AFM images are intended only to visualize species present at different time points of protein aggregation and do not aim to quantify the relative abundance of both DO and RO.

**Fig. 1:**
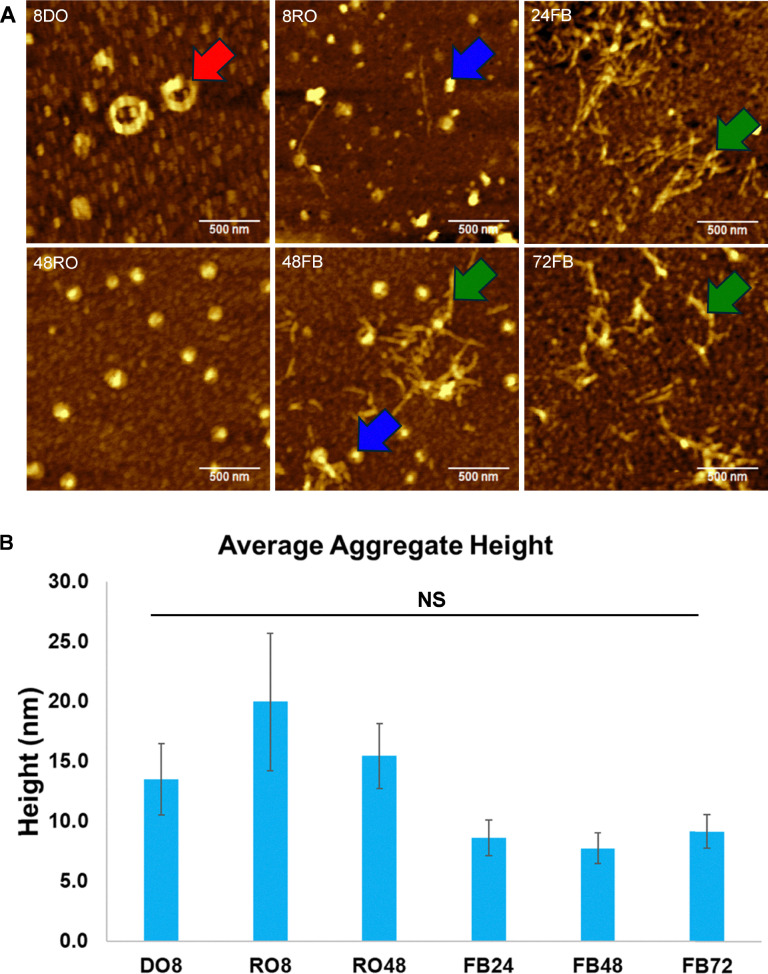
AFM images (A) and histograms (B) of protein aggregates observed during the onset (8 h), middle (24 h), and at the end (48 h and 72 h) of 0N4R Tau aggregation. Red arrows indicate DO, blue arrows indicate RO, and green arrows indicate fibrils. Scale bars are 500 nm. For the reported height profiles, 20–30 individual protein species were analyzed. For every sample, 2–3 individual AFM images were recorded. Error bars represent standard deviations. NS – non-statistical significance.

Next, we acquired nano-IR spectra of all protein aggregates discussed above. The spectra exhibited both amide I (1615–1700 cm^−1^) and II (∼1550 cm^−1^) bands. Amide I bands primarily originate from the C=O vibration of the peptide (amide) bond and can, therefore, be used to interpret the secondary structure of protein aggregates. Expanding upon this, we fit the amide I band in the acquired spectra to reveal the relative contributions of parallel β-sheets, disordered proteins, and antiparallel β-sheets to the secondary structure.

We found that both RO and DO observed during the onset of protein aggregation had similar secondary structures dominated by parallel and antiparallel β-sheets (∼60%) with ∼40% unordered protein (Fig. 2B). We also observed no evolution in the protein secondary structure between 8 h and 48 h RO. The same conclusion could be made about fibrillar species observed at the middle (24 h) and late (48 and 72 h) stages of Tau aggregation. Thus, these findings indicate that although 0N4R Tau forms morphologically different oligomers at the early stages of protein aggregation, two oligomer populations display similar average secondary structures within the resolution of the current analysis. We also found that both oligomers and fibrils share strong similarities with the secondary structure of 0N4R Tau fibrils.

**Fig. 2:**
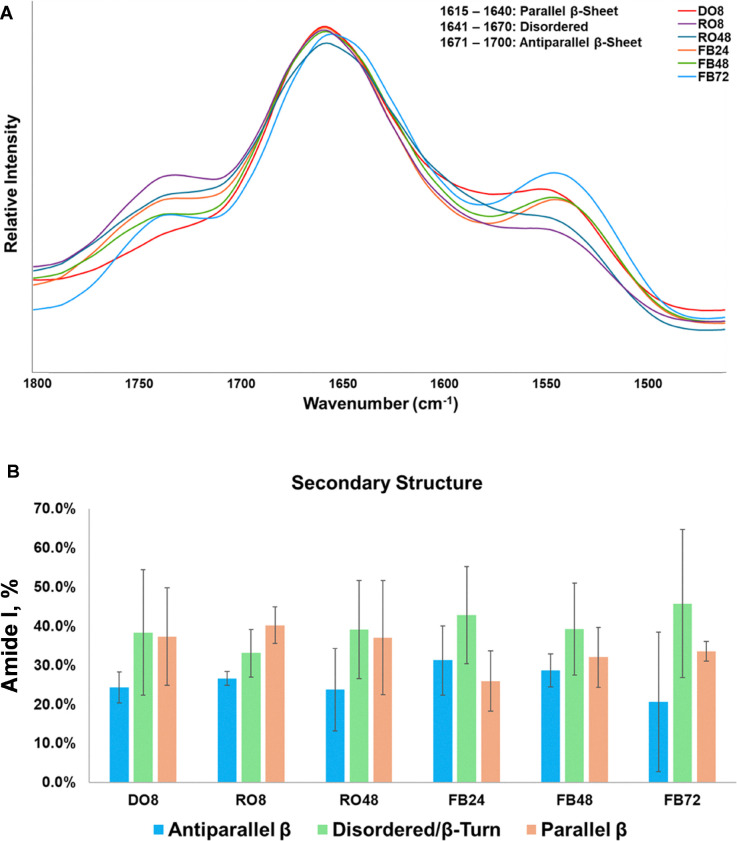
(A) AFM-IR spectra acquired from 0N4R Tau aggregates observed during the onset (8 h), middle (24 h), and at the end (48 h and 72 h) of protein aggregation. (B) Histogram of amide I fit indicating the relative contributions of parallel β-sheets, disordered proteins, and antiparallel β-sheets to the secondary structure of 0N4R aggregates. Statistical analysis showed no statistical significance between the relative contributions of parallel β-sheets, disordered proteins, and antiparallel β-sheets to the secondary structure of 0N4R aggregates observed at different time points of protein aggregation.

Based on the reported results, one can question the potential of AFM-IR in the structural analysis of such aggregates. However, previously reported results by Warren and co-workers demonstrated that RO and DO formed by hIAPP exhibited drastically different IR spectra, which allowed them to resolve their differences in their secondary structures.^39^ Based on this evidence, we can conclude that 0N4R Tau indeed formed structurally similar, if not identical, DO and RO at the early stage of protein aggregation. Nevertheless, morphological analysis of 0N4R Tau aggregation allowed us to conclude that DO rather than RO triggered fibril formation. It should be noted that Warren and co-workers also concluded that DO observed upon hIAPP aggregation seeded fibrils.^39^ Based on these findings, we can conclude that both hIAPP and 0N4R Tau exhibit DO and RO, with DO being “on-path” and RO likely being “off-path” species in protein aggregation. Certainly, analysis of the aggregation dynamics of other proteins like TDP-43 and Tau isoforms with 1N and 2N repeats is required to understand whether the formation of DO and RO is a general phenomenon observed across a large group of amyloidogenic proteins.

In summary, our results show that during the initial phases of 0N4R Tau protein aggregation, two distinct morphological species emerge: DO and RO (Fig. 3). As the process advances, DO vanish as fibrillar structures develop, whereas RO persist into the later stages. This suggests that DO are “on-pathway” intermediates that directly transition into fibrils, while RO represent “off-pathway” structures that do not evolve into larger supramolecular assemblies.

**Fig. 3:**
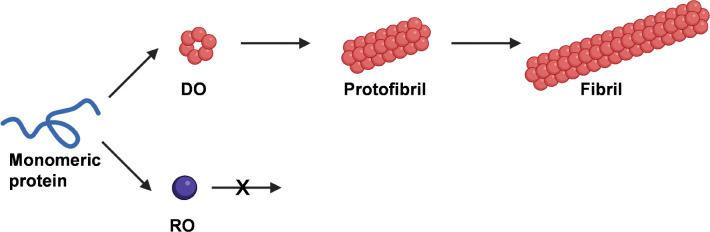
Schematic illustration of “on-path” (red) and “off-path” (purple) aggregation of monomeric 0N4R Tau.

Despite their different fates, both DO and RO species possess similar secondary structures, primarily composed of β-sheets with approximately 40% unordered regions. These findings highlight the intricate nature of Tau aggregation, a factor that is vital for developing targeted inhibitors designed to block the pathological conversion of oligomers into fibrils.

Finally, we note that Tau exhibits a large number of post-translational modifications (PTMs), including phosphorylation.^43^ One can expect that such PTMs could alter the mechanisms of protein self-assembly.^44^ Therefore, in our future studies, we plan to examine the effects of PTMs on the self-assembly of Tau proteins.

The manuscript was written through the contributions of all authors. All authors have given approval to the final version of the manuscript.

## Conflicts of interest

There are no conflicts to declare.

## Supplementary Material

CC-062-D6CC02327D-s001

## Data Availability

All data are available from the lead contact upon reasonable request. Supplementary information: supplemental figures and experimental procedures can be found in the supplementary information (SI). See DOI: https://doi.org/10.1039/d6cc02327d.

## References

[cit1] McLean C. A., Cherny R. A., Fraser F. W., Fuller S. J., Smith M. J., Beyreuther K., Bush A. I., Masters C. L. (1999). Ann. Neurol..

[cit2] Gouras G. K., Tampellini D., Takahashi R. H., Capetillo-Zarate E. (2010). Acta Neuropathol..

[cit3] Goedert M., Spillantini M. G., Jakes R., Rutherford D., Crowther R. A. (1989). Neuron.

[cit4] Himmler A., Drechsel D., Kirschner M. W., Martin, Jr. D. W. (1989). Mol. Cell. Biol..

[cit5] Weingarten M. D., Lockwood A. H., Hwo S. Y., Kirschner M. W. (1975). Proc. Natl. Acad. Sci. U. S. A..

[cit6] Grundke-Iqbal I., Iqbal K., Tung Y. C., Quinlan M., Wisniewski H. M., Binder L. I. (1986). Proc. Natl. Acad. Sci. U. S. A..

[cit7] Alonso Adel C., Mederlyova A., Novak M., Grundke-Iqbal I., Iqbal K. (2004). J. Biol. Chem..

[cit8] Alonso A. D., Zaidi T., Novak M., Barra H. S., Grundke-Iqbal I., Iqbal K. (2001). J. Biol. Chem..

[cit9] Giamblanco N., Fichou Y., Janot J. M., Balanzat E., Han S., Balme S. (2020). ACS Sens..

[cit10] Falcon B., Zivanov J., Zhang W., Murzin A. G., Garringer H. J., Vidal R., Crowther R. A., Newell K. L., Ghetti B., Goedert M., Scheres S. H. W. (2019). Nature.

[cit11] Zhang W., Falcon B., Murzin A. G., Fan J., Crowther R. A., Goedert M., Scheres S. H. (2019). eLife.

[cit12] Ait-Bouziad N., Lv G., Mahul-Mellier A. L., Xiao S., Zorludemir G., Eliezer D., Walz T., Lashuel H. A. (2017). Nat. Commun..

[cit13] Takashima A. (2013). J. Alzheimer's Dis..

[cit14] Ali A., Holman A. P., Rodriguez A., Matveyenka M., Kurouski D. (2024). Protein Sci..

[cit15] Ali A., Matveyenka M., Pickett D. N., Rodriguez A., Kurouski D. (2025). J. Neurochem..

[cit16] Lasagna-Reeves C. A., Castillo-Carranza D. L., Sengupta U., Sarmiento J., Troncoso J., Jackson G. R., Kayed R. (2012). FASEB J..

[cit17] Karikari T. K., Nagel D. A., Grainger A., Clarke-Bland C., Hill E. J., Moffat K. G. (2019). Anal. Biochem..

[cit18] Eisenberg D. S., Sawaya M. R. (2017). Nature.

[cit19] Shafiei S. S., Guerrero-Munoz M. J., Castillo-Carranza D. L. (2017). Front. Aging Neurosci..

[cit20] Nath S., Agholme L., Kurudenkandy F. R., Granseth B., Marcusson J., Hallbeck M. (2012). J. Neurosci..

[cit21] Rajendran L., Honsho M., Zahn T. R., Keller P., Geiger K. D., Verkade P., Simons K. (2006). Proc. Natl. Acad. Sci. U. S. A..

[cit22] Wang Y., Balaji V., Kaniyappan S., Kruger L., Irsen S., Tepper K., Chandupatla R., Maetzler W., Schneider A., Mandelkow E., Mandelkow E. M. (2017). Mol. Neurodegener..

[cit23] Clavaguera F., Bolmont T., Crowther R. A., Abramowski D., Frank S., Probst A., Fraser G., Stalder A. K., Beibel M., Staufenbiel M., Jucker M., Goedert M., Tolnay M. (2009). Nat. Cell Biol..

[cit24] LaPointe N. E., Morfini G., Pigino G., Gaisina I. N., Kozikowski A. P., Binder L. I., Brady S. T. (2009). J. Neurosci. Res..

[cit25] Morfini G. A., Burns M., Binder L. I., Kanaan N. M., LaPointe N., Bosco D. A., Brown, Jr. R. H., Brown H., Tiwari A., Hayward L., Edgar J., Nave K. A., Garberrn J., Atagi Y., Song Y., Pigino G., Brady S. T. (2009). J. Neurosci..

[cit26] Centrone A. (2015). Annu. Rev. Anal. Chem..

[cit27] Kurouski D., Dazzi A., Zenobi R., Centrone A. (2020). Chem. Soc. Rev..

[cit28] Ramer G., Ruggeri F. S., Levin A., Knowles T. P. J., Centrone A. (2018). ACS Nano.

[cit29] Dazzi A., Prater C. B. (2017). Chem. Rev..

[cit30] Mathurin J., Dartois E., Pino T., Engrand C., Duprat J., Deniset-Besseau A., Borodnics F., Sandt C., Dazzi A. (2019). Astron. Astrophys..

[cit31] Ruggeri F. S., Mannini B., Schmid R., Vendruscolo M., Knowles T. P. J. (2020). Nat. Commun..

[cit32] Ruggeri F. S., Vieweg S., Cendrowska U., Longo G., Chiki A., Lashuel H. A., Dietler G. (2016). Sci. Rep..

[cit33] Zhaliazka K., Kurouski D. (2023). Nanoscale.

[cit34] Zhou L., Kurouski D. (2020). Anal. Chem..

[cit35] Dou T., Kurouski D. (2022). ACS Chem. Neurosci..

[cit36] Dou T., Li Z., Zhang J., Evilevitch A., Kurouski D. (2020). Anal. Chem..

[cit37] Ruggeri F. S., Longo G., Faggiano S., Lipiec E., Pastore A., Dietler G. (2015). Nat. Commun..

[cit38] Zhaliazka K., Kurouski D. (2022). ACS Chem. Neurosci..

[cit39] Warren D., Sitton J., Kurouski D. (2026). Protein Sci..

[cit40] Ali A., Matveyenka M., Rodriguez A., Kurouski D. (2024). J. Phys. Chem. Lett..

[cit41] Blurton-Jones M., Laferla F. M. (2006). Curr. Alzheimer Res..

[cit42] Gerson J., Castillo-Carranza D. L., Sengupta U., Bodani R., Prough D. S., DeWitt D. S., Hawkins B. E., Kayed R. (2016). J. Neurotrauma.

[cit43] Skrehot J. T., Kurouski D. (2026). Protein Sci..

[cit44] Pillai V. V. S., Smeding T., Meng J. X., Klenerman D., Ruggeri F. S. (2025). ACS Meas. Sci. Au.

