# Supplementary material for: 0N4R Tau aggregates producing morphologically different and structurally similar “on-path” and “off-path” oligomers
Source: Chem Commun (Camb). 2026 Jul 9;62(58):14602–5. doi: 10.1039/d6cc02327d (PMC13347184; doi:10.1039/d6cc02327d)
Supplement: CC-062-D6CC02327D-s001 [file CC-062-D6CC02327D-s001.pdf]

Supplementary Information (SI) for ChemComm.
This journal is © The Royal Society of Chemistry 2026

0N4R Tau Aggregates Producing Morphologically Different and Structurally Similar "On-Path" and "Off-Path" Oligomers

Joshua Skrehot,[1] Mikhail Matveyenka,[1] and Dmitry Kurouski*[1,2]

1. Department of Biochemistry and Biophysics, Texas A&M University, College Station, Texas 77843, United States
2. Department of Biomedical Engineering, Texas A&M University, College Station, Texas, 77843, United States

Email: dkurouski@tamu.edu

Supporting Information

Methods:

Protein expression and purification: 0N4R Tau was expressed in *Escherichia coli* BL21 (DE3) Rosetta strain incubated in LB broth media. To activate protein expression, 1 mM Isopropyl β-D-1-thiogalactopyranoside (IPTG) was used. Next, bacterial cultures were centrifuged at 8,000 RPM for 10 minutes. The formed pellet was re-suspended in lysis buffer consisting of 8M Urea, 50 mM Tris-HCl, 300 mM NaCl, pH 8.0 that contained 1 mM phenylmethylsulfonyl fluoride (PMSF). Next, the resuspended pellet underwent 5 cycles of freeze-thaw followed by sonication. After that, samples were centrifuged at 16,000 g for 1 h to separate the soluble fraction. Next, supernatants were passed through a syringe filter with a pore size of 0.4 µm and processed on a gravity column equipped with Ni-NTA agarose beads. 0N4R protein was eluted from the column using an elution buffer containing 50 mM Tris-HCl, 300 mM NaCl, and 300 mM imidazole. The eluted protein fractions were dialyzed against 10 mM Tris buffer (pH 7.4) using a dialysis membrane with a molecular weight cut-off of 30 kDa to remove urea and imidazole. After dialysis, protein samples were concentrated using centrifugal concentrators with a 10 kDa molecular weight cut-off. The final concentration of the purified protein was approximately 2.5 mg/ml. Sodium Dodecyl Sulfate Polyacrylamide Gel Electrophoresis (SDS-PAGE) analysis was conducted to assess the purity and size of the obtained proteins.

Tau aggregation: 30 µM of 0N4R Tau was dissolved in 10 mM Tris buffer (pH 7.4). The samples were dispensed into 1ml Eppendorf tubes kept at 37°C for 72 h under 510 rpm agitation.

Kinetic measurements: Rates of protein aggregation were measured using a thioflavin T (ThT) fluorescence assay. For this, samples were mixed with 2 mM of ThT solution and placed into 96 well-plate that was kept in the plate reader (Tecan, Männedorf, Switzerland) at 37 °C for 72 h under 510 rpm agitation. Fluorescence measurements were taken every 10 min (excitation 450 nm; emission 488 nm). Each kinetic curve shown in Figure S1 is an average of three independent measurements.

Atomic force microscopy-Infrared spectroscopy (AFM-IR): Imaging and spectral analysis were obtained using a nanoIR3 system (Bruker, Santa Barbara, CA, USA), equipped with a QCL laser. ContGB-G AFM probes specific to contact mode were utilized with probe parameters: frequency of 13 kHZ, spring constant of 0.2 N/m, and a length of 450 µm. The tip was optimized using a polymethyl acrylate standard for the wavenumbers: 1400-1800 cm^{-1}. Laser parameters include a power of 25.49%, polarization at 90 degrees, IR focus of 70456, and a pulse rate around 828 kHz. Image dimensions of 1-10 µm were acquired at a scan rate of 0.5-0.8 Hz, an I and P gain ranging from 1/2 to 5/10, and a resolution of 256 for both the X and Y. A total of 30 spectra per sample were obtained with a co-average of 3 for each spectrum at a spectral resolution of 2 cm^{-1}/pt. In each sample, ~30 individual aggregates were analyzed. Protein samples were first deposited onto a 70 nm gold-coated silicon wafer at a volume of 2.5 µL, left to dry at room temperature for roughly 15 minutes or until a visible coffee ring under the drop is present, then rinsed with distilled water and dried using a N_2 air flow. Spectral processing was conducted using MATLAB, equipped

with a PLS Toolbox version 9.0 (Eigenvector Research, Inc., Manson, WA). Spectra are first applied a smoothing processing of Savitzky-Golay at a polynomial order of 0, area normalized, and baselined with automated weighted least squares. After processing, spectra were then peak fitted using GRAMS/AI Spectroscopy Software to assign peaks respective to the secondary structure of the protein.

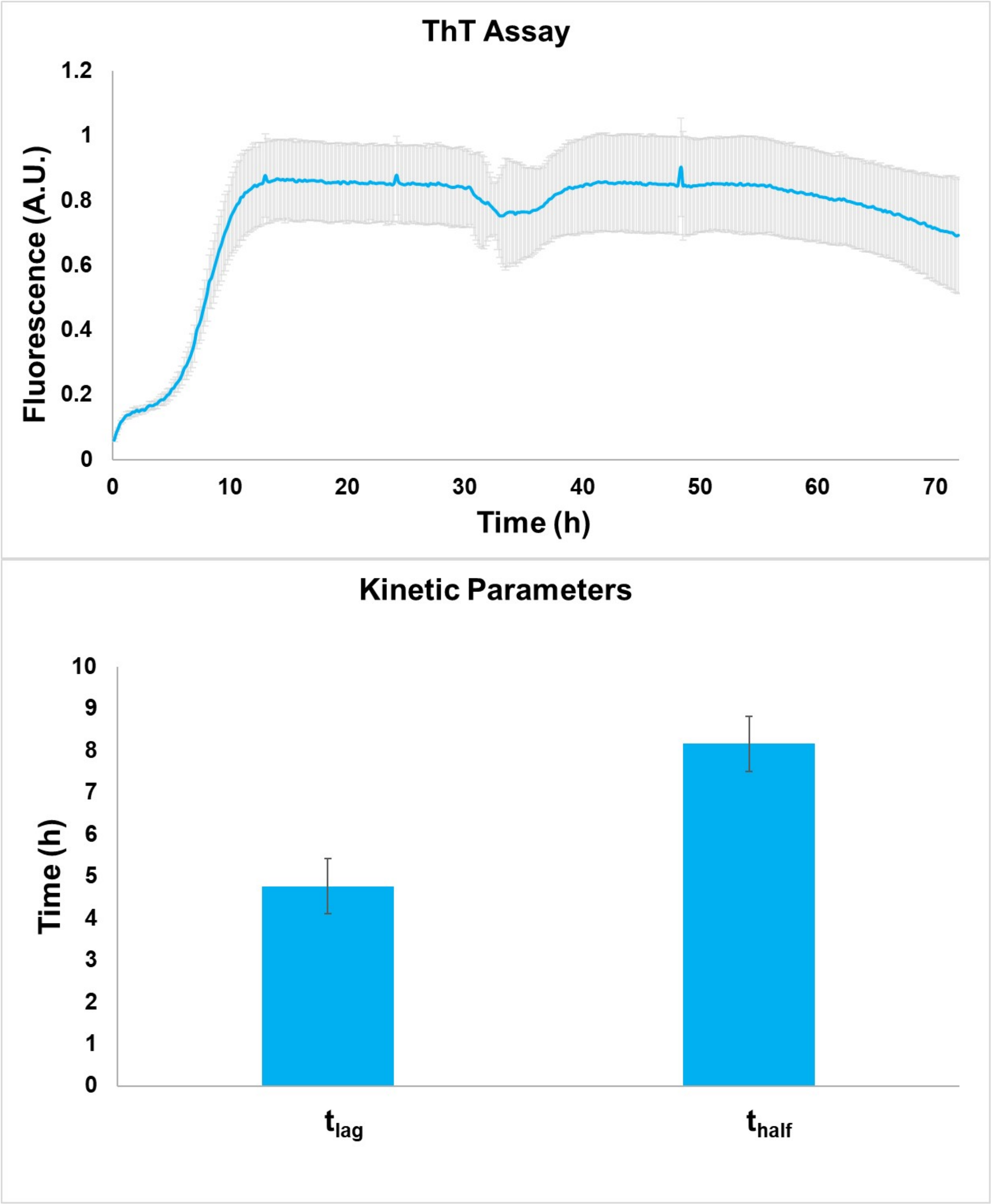

Figure F1. ThT kinetics (top) and corresponding histograms of t_{lag} (10% of ThT intensity) and t_{half} (50% of ThT intensity) of 0N4R Tau aggregation.
